# Feasibility of Three Alcohol Brief Intervention Intensities in Sri Lankan Primary Care: A Pilot Trial Including Family Member Outcomes

**DOI:** 10.1192/bjo.2026.11556

**Published:** 2026-06-30

**Authors:** Dewasmika Ariyasinghe, Thilini Rajapakse, Sally Carter, Grace Joshy, Kamalini Lokuge

**Affiliations:** 1 Australian National University, Canberra, Australia; 2 University of Peradeniya, Peradeniya, Sri Lanka; 3 Teaching Hospital Peradeniya, Peradeniya, Sri Lanka

## Abstract

**Aims:**

Risky drinking causes substantial harm in Sri Lanka, yet despite WHO endorsement, brief interventions remain unevaluated in primary care settings. Sri Lanka’s outpatient departments record 55 million visits annually, representing an untapped opportunity for systematic opportunistic alcohol intervention delivery. This pilot randomized trial aimed to assess feasibility and acceptability of three brief intervention intensities, evaluate appropriateness of outcome measures including an innovative family member-reported burden measure, and generate preliminary data to inform a future adequately powered effectiveness trial.

**Methods:**

One hundred and fifty adult male risky drinkers (AUDIT-C ≥4) presenting to the outpatient department of Teaching Hospital Peradeniya were randomized equally to three arms representing increasing intensity along a brief intervention continuum: Feedback on AUDIT-C (FOA, approximately 3 minutes), Unit of Alcohol education (UOA, approximately 5 minutes), or Adapted Brief Intervention (ABI, 15-30 minutes). Trained non-specialist research assistants delivered all interventions and followed up at six months. Feasibility was assessed through recruitment rate, retention at 6-month follow-up, and participant-reported acceptability. Drinking-related outcomes included AUDIT-C scores, binge drinking frequency, and Alcohol Problems Questionnaire (APQ) scores. Family burden was measured using the AFI-Net Family Member Questionnaire.

**Results:**

Target recruitment (n=50 per each arm) was achieved within approximately six weeks. Patient retention at 6-month follow-up was 92%, 84% and 90% for FOA, UOA and ABI arms respectively. Family member retention was 93.7% (74/79 enrolled), demonstrating feasibility of this novel dyadic design. All questionnaires were comprehensible and outcome measurement was feasible. Patient feedback was overwhelmingly positive across all three arms. Of the 133 who came for follow-up 111 (83.4%) were in precontemplation, at recruitment. Baseline scores in all outcome measures were comparable between the three arms. Pilot data indicate improvements in all outcomes in all three arms–change from baseline (mean±SD) in AUDIT-C score were −0.80±1.94, −1.55±2.50, and −2.36±2.81; in binge drinking days per month −1.99±6.20, −1.75±6.17 and −3.75±9.52; APQ score −2.23±5.84, −2.24±4.14 and −2.45±4.37; and family burden −3.65±6.37, −1.3±8.37 and −1.8±9.39, for FOA, UOA and ABI arms respectively.

**Conclusion:**

This is the first alcohol brief intervention pilot trial conducted in Sri Lankan primary care. It is, to the best of our knowledge, also the first brief intervention study internationally to measure family member-reported burden as a primary outcome. All three intervention intensities proved feasible and acceptable in a high-volume, resource-limited outpatient setting. The observed dose-response pattern in patient-reported outcomes across intervention intensities is promising but warrants confirmation in an adequately powered efficacy trial.

